# Review of Gallium-68 PSMA PET/CT Imaging in the Management of Prostate Cancer

**DOI:** 10.3390/diagnostics8010016

**Published:** 2018-02-11

**Authors:** Nat P. Lenzo, Danielle Meyrick, J. Harvey Turner

**Affiliations:** 1 Nuclear Oncology, Theranostics Australia, 106/1 Silas Street, Richmond Quarter Building, East Fremantle, WA 6158, Australia; danielle.meyrick@theranostics.com.au; 2 School of Medicine and Pharmacology, University of Western Australia, 35 Stirling Highway, Nedlands, WA 6009, Australia; harvey.turner@health.wa.gov.au

**Keywords:** theranostics, nuclear oncology, molecular imaging, ^68^Ga-PSMA PET/CT, prostate cancer

## Abstract

Over 90% of prostate cancers over-express prostate specific membrane antigen (PSMA) and these tumor cells may be accurately targeted for diagnosis by ^68^Ga-PSMA-positron emission tomography/computed tomography (^68^Ga-PSMA-PET/CT) imaging. This novel molecular imaging modality appears clinically to have superseded CT, and appears superior to MR imaging, for the detection of metastatic disease. ^68^Ga-PSMA PET/CT has the ability to reliably stage prostate cancer at presentation and can help inform an optimal treatment approach. Novel diagnostic applications of ^68^Ga-PSMA PET/CT include guiding biopsy to improve sampling accuracy, and guiding surgery and radiotherapy. In addition to facilitating the management of metastatic castrate resistant prostate cancer (mCRPC), ^68^Ga-PSMA can select patients who may benefit from targeted systemic radionuclide therapy. ^68^Ga-PSMA is the diagnostic positron-emitting theranostic pair with the beta emitter Lutetium-177 PSMA (^177^Lu-PSMA) and alpha-emitter Actinium-225 PSMA (^225^Ac-PSMA) which can both be used to treat PSMA-avid metastases of prostate cancer in the molecular tumor-targeted approach of theranostic nuclear oncology.

## 1. Introduction

Gallium-68-prostate specific membrane antigen (^68^Ga-PSMA) positron emission tomography/computed tomography (PET/CT) imaging is revolutionizing the management of prostate cancer since its advent in 2013 [1]. Within 1 year, centers in every state in Australia were performing ^68^Ga-PSMA PET/CT and in less than 4 years, this form of targeted molecular imaging is not only being incorporated into routine clinical management of prostate cancer patients serviced by 40 centers country-wide, but arguably has become the standard of care throughout Australia in the diagnosis, selective staging and monitoring of therapeutic response of prostate cancer. Apart from the significantly higher diagnostic sensitivity that ^68^Ga-PSMA PET/CT offers, it also constitutes the diagnostic complementing the ^177^Lutetium-PSMA [2] and more recently the ^225^Actinium-PSMA [3], theranostic pairs, both currently being investigated for the therapy of metastatic castrate resistant prostate cancer (mCRPC). Why and how did this revolutionizing phenomenon occur in Australia and what significance does it have for oncological clinical practice throughout the world?

Australia has a record of early adoption of novel imaging technology. Within 4 months of Roentgen’s discovery of X-rays in Germany in November 1895, Walter Drowley Filmer, an amateur scientist and electrician, used X-rays to localize a foreign object within a patient in Newcastle, New South Wales [4]. The introduction of CT scans took a little longer with Australia waiting 2 years from the installation of the first whole body CT scanner at Northwick Park Hospital in London in 1975 [5] to the installation of the first CT scanner in a Sutherland hospital in Sydney [6]. The first human magnetic resonance imaging (MRI) scan was performed in 1977 and in 1986 the first MRI was installed in the private sector, in Sydney [7].

Cancer is a molecular disease and the advent of theranostic nuclear oncology is supplanting the traditional anatomic and functional imaging approaches typified by CT and MRI. Cancer is a disease of uncontrolled growth and proliferation whereby cells have escaped the body’s normal growth control mechanisms and have gained the ability to divide indefinitely. It is a multi-step evolutionary process that requires the accumulation of multiple genetic changes over time. Cell-signaling pathways, both external, and internal, are altered, driving the proliferation of neoplastic cells. We are beginning to understand the underlying genomics and proteomics underpinning neoplastic transformation and are now altering our treatment strategies to target and mitigate the changes driving tumor growth and development. Thus the assessment of molecular receptors such as human epidermal growth factor receptor 2 (HER2) in breast cancer and epidermal growth factor receptor (EGFR) in non-small cell lung cancer is now routine to guide treatment regimens [8]. The overexpression of PSMA in prostate cancer, which in cell lines has been demonstrated to increase angiogenesis, and enhance metabolism of polyglutamated folates and uptake of monoglutamated folates, thus imparting a proliferative advantage [9], may be similarly exploited.

Despite the move toward molecular diagnostics, our clinical imaging paradigms for diagnosing cancer and for monitoring cancer therapy have largely remained at the anatomical rather than the cellular or molecular level. We continue to use Response Evaluation Criteria in Solid Tumors (RECIST) criteria [10] based on anatomical size for monitoring treatment response even though, as we now appreciate, measurement of tumor diameter is at best a crude instrument for understanding what is happening at a cellular level within the tumor.

Prostate cancer is one of the leading causes of morbidity and death in the Western world. It is the second most common cancer in men worldwide [11]. With the aging population more men are being diagnosed with prostate cancer, as has been demonstrated in the UK with a 44% increase in prostate cancer incidence rates since the early 1990’s [12]. In Australia and the UK, more men now die from prostate cancer each year than women die of breast cancer [13]. Unlike in breast cancer and other tumor types such as colon cancer, there is no well-defined screening program for prostate cancer apart from the somewhat controversial use of prostate specific antigen (PSA) as a potential screening tool [14,15,16]. Also, unlike the major advances seen with treatments in breast cancer and colon cancer relatively little progress has been made in patients with prostate cancer which has spread beyond the confines of the prostate gland at the time of surgery. The failure rate after primary therapy for prostate cancer is between 20–60% of patients treated [17,18] and the 5-year survival in patients presenting with high volume metastatic disease is less than 30% [19].

The mainstays of diagnostic investigations for over 30 years have been physical examination (i.e., the digital rectal examination—DRE), blood investigation in the form of PSA and biopsy, be it trans-rectal, trans-perineal, blind or image-directed. Imaging investigations, predominantly CT and more recently MRI, and in particular multiparametric MRI, have contributed additional value to the diagnostic algorithm [20,21]. Occasionally bone scintigraphy with technetium-99 based compounds may allude to, or increase the likelihood of, a diagnosis of prostate cancer, particularly in the setting of an incidental high PSA result in an otherwise unsuspecting patient. Ultimately, the diagnosis is established by pathological confirmation, most commonly from prostatic biopsy, but occasionally from biopsy material obtained from extra-prostatic metastatic deposits.

Diagnosis by DRE, PSA and anatomical imaging has a number of flaws. The positive predictive value of DRE, in settings of low PSA, has been shown to be 5–30% [22]. DRE not only misses a number of clinically important prostate cancers but also has a high false positive rate [23]. PSA is a non-specific blood marker and may be raised in non-malignant clinical scenarios such as prostatitis and benign prostatic hypertrophy. Furthermore, a low PSA does not necessarily exclude the presence of prostatic malignancy [24]. Anatomical imaging with CT has poor sensitivity and specificity within the prostate gland itself and has been used predominantly to stage the disease after a diagnosis has been made. CT may show the evidence of metastatic spread to pelvic lymph nodes, or the seminal vesicles, and occasionally demonstrates sclerotic deposits characteristic of the osseous metastases from prostate adenocarcinoma. Unfortunately, CT can only give a suggestion of potential metastatic disease based on changes to anatomy, particularly with regard to size, rather than giving information germane to actual tumor involvement of a tissue. This limitation in early stage disease is well documented. A recent meta-analysis of 24 studies showed an acceptable specificity of 82% for lymph node diagnosis but an unacceptable sensitivity of only 42% with CT [25].

Multiparametric MRI is being utilized with increasing frequency worldwide due to its higher sensitivity, specificity and predictive value in assessing the prostate gland [21,26]. Apart from detecting changes in prostatic architecture and anatomy, this technique gives insights into potential malignant transformation via parameters such as diffusion restriction. The use of multiparametric MRI also appears to be increasing the proportion of clinically relevant diagnosed prostate cancers [27]. This technique is also more accurate than CT in assessing the lymph nodes within the pelvis [28]. However MRI, although a major step forward, is limited by issues such as claustrophobia, cost and the field of view limited usually to the pelvis alone.

In the 1990’s attempts were made to specifically target tumor characteristics with molecular imaging techniques based upon single photon emission computed tomography (SPECT) and positron emission tomography (PET). The objective was to improve the accuracy of detecting prostate cancer at an earlier time point than with anatomical imaging techniques such as CT. Prostate cancer cells overexpress a surface marker known as Prostate Specific Membrane Antigen (PSMA). Antibodies were initially developed, followed by peptides, to target this antigen as the basis for prostate cancer specific molecular imaging agents.

PSMA is a 750-amino acid transmembrane protein. In benign prostatic tissue, it is found within the apical epithelium of secretory ducts. While the physiologic role of PSMA in the prostate remains unclear, its enzymatic role is in the cleavage of α-linked glutamate from *N*-acetylaspartyl glutamate and γ-linked glutamates from polyglutamated folates [9]. During malignant transformation PSMA is translocated to the luminal surface of the ducts [29]. There an overexpression is seen. This overexpression has not been found in benign disease such as prostatic hyperplasia [30]. Several functions, including roles in cell migration, cellular nutrition, transport, and signal transduction, have been ascribed to PSMA [31]. Once a ligand binds to the PSMA protein it is internalized into the cell. However, PSMA is not prostate specific as receptors are found within lacrimal and salivary glands, the kidneys, small intestine, liver and spleen [32]. It is also expressed in tumor associated angiogenesis, and has been described in other tumors, such as glioblastoma, thyroid cancer, gastric, breast, renal and colorectal cancers [32]. 

Despite its presence in other tissues, PSMA has been found to be an excellent agent for targeted imaging and therapy. It is overexpressed one-hundred to one thousand-fold in 95% of prostate cancer cells [32]. The PSMA-ligand complex is internalized after binding via clathrin coated pits and endosome accumulation [33], which leads to enhanced retention important for both image quality and therapeutic efficacy. PSMA expression appears to correlate with advanced disease, castrate resistant disease, Gleason score and PSA level [34,35].

A variety of monoclonal antibodies (mAb) have been developed targeting both the intracellular and extracellular epitopes of PSMA. The typical issues related to antibodies, such as long circulating half-life, background activity and poor target tissue uptake have impeded commercial development of antibody based imaging and therapeutic agents. Despite this, two mAb variants have demonstrated high affinity, and specific and efficient targeting in vivo. These include the murine mAb 7E11, which binds an intracellular domain of PSMA, and the humanized mAb hJ591, which binds to an extracellular domain of PSMA [36]. ^111^Indium-7E11 has been investigated clinically as a SPECT imaging agent for recurrent and metastatic prostate cancer [37,38] (^111^In-7E11, ProstaScint™). The therapeutic concomitant ^90^Yttrium radiolabelled conjugate has also been studied [39]. But the high myelotoxic rate seen with the Y-90 conjugate also limited further development. Improvements in other imaging modalities and the overall poor sensitivity has lead to the virtual demise of ProstaScint™ as an imaging tool in most parts of the world. The J591 mAb has been clinically investigated for PET/CT imaging as ^89^Zr-hJ591 [40] and for therapy as ^177^Lu-hJ591 [41] and clinical development of radioimmunotherapy of mCRPC continues.

Small molecule PSMA-peptide inhibitor (also known as ligand) molecules have been developed which show high binding and these are the mainstay of current PSMA imaging techniques. Several PSMA ligands, differing slightly in chemical structure, are commercially available. Three peptide ligands have become the dominant agents in clinical use; one of these may be labelled only with ^68^Ga, while two may be labelled with either ^68^Ga or ^177^Lu, allowing, where desired, a theranostic approach to prostate cancer. A rational approach has underpinned the development of these agents, with PSMA affinity (and therefore tumor uptake) and rapid blood clearance being key parameters targeted.

Glu–NH–CO–Lys–(Ahx)–[^68^Ga-HBED-CC] (HBED CC: N,N′-Bis(2-hydroxy-5-(ethylene-beta-carboxy)benzyl)ethylenediamine N,N′-diacetic acid (known also as ^68^Ga-PSMA-11) is perhaps the most widely used agent for prostate cancer PET/CT imaging. The HBED chelator forms a thermodynamically stable complex with Ga, and ^68^Ga-PSMA-11 shows fast blood clearance, low liver uptake and high uptake in PSMA-expressing tissues [40]. The PSMA-617 agent, a 1,4,7,10-tetraazacyclododecane-1,4,7,10-tetraacetic acid (DOTA) conjugated molecule suitable for labelling with ^68^Ga and ^177^Lu, allows the use of a theranostic pair. Numerous studies have evaluated the role of this agent in both imaging and therapy; some have found that this, and other DOTA-conjugated agents, have lower tumor uptake relative to HBED-CC chelated agents [42] although the agent is commonly used due to its possible therapeutic applications. The lower tumor affinity of DOTA-conjugated compounds led to the development of 1,4,7,10-tetraazacyclododececane,1-(glutaric acid-4,7,10-triacetic acid (DOTAGA)-conjugated compounds [43], including PSMA-I&T, which also incorporates a lipophilic peptidic linker to improve the interaction with PSMA, and therefore enhance tumor uptake. This agent, like PSMA-617, allows a theranostic approach to prostate cancer diagnosis and treatment. The clinical efficacy of ^68^Ga-PSMA-I&T appears equivalent to ^68^Ga-PSMA-HBED though imaging characteristics appear slightly better for the latter imaging agent [44].

Technetium [45], iodine [46] and fluorine-18 [47,48,49] labelled PSMA analogues have also been developed, and although potentially promising, particularly due to their longer half-life, this paper will concentrate on the Gallium-68 agents which could be considered now almost part of routine clinical practice in Australia. F-18 labelled PSMA ligands have recently been reviewed elsewhere [50,51]. Choline, though initially considered a potential diagnostic tool [52], although established and reimbursed in Europe, has not been well established in Australia and may now possibly be relegated to the history books with the advent of ^68^Ga PSMA PET/CT [51,53,54]. 

A clear advantage of ^68^Ga-labelled diagnostic radiopharmaceuticals is on-site synthesis, with gallium-68 produced and eluted from a ^68^Ge/^68^Ga generator. This process is analogous to the ^99^Mo/^99m^Tc generator, the “workhorse” of nuclear medicine for the past 50 years. In-house synthesis allows, within the limits of ^68^Ge decay and ^68^Ga ingrowth, on demand radiopharmaceutical production, without the need for a medical cyclotron and its supporting infrastructure. These benchtop generators have a footprint such that they can be easily housed in most imaging facility supporting laboratories without significant, if any, modification to infrastructure. In addition, ongoing developments by the various manufacturers mean that they are becoming increasingly user-friendly, with manufacturer efforts focusing, importantly, on radiation safety.

There are now several GMP-compliant ^68^Ge/^68^Ga generators available which yield highly chemically pure ^68^Ga on elution, with minimal breakthrough of parent ^68^Ge and other metal impurities [55], which may interfere significantly with peptide radiolabelling. These generators differ principally in the stationary phase to which the parent radioisotope (^68^Ge) is adsorbed. This phase determines the concentration, ranging from 0.05 to 1.0 M, of HCl mobile phase that must be used to separate daughter ^68^Ga from ^68^Ge during elution. In turn this mobile phase has an effect on the chemistry of radiolabelling. The various approaches to elution and subsequent chemistry of radiolabelling have been nicely summarized by Mueller et al. [56].

PSMA labelling with ^68^Ga is most often performed with modular synthesis units; these use sterile, single use cassettes and GMP compliant reagents and are accommodated in a hot cell, or similarly shielded workspace. Such units have the advantage of automation and minimal operator radiation exposure, but have lengthy synthesis times (up to 35 min), during which period, of course, ^68^Ga decays, meaning that the efficiency of the generator, in terms of patient doses produced, is decreased. Furthermore, these units are costly, and the design of some is not failsafe, leading to unsuccessful syntheses, and consequences in terms of workflow, patient scheduling and inconvenience.

Recently, several one- or two-step GMP compliant kit-based synthesis cold kits have become available. While these are not compatible with all ^68^Ge/^68^Ga generators, they have short reaction times at room temperature, with minimal risk of operator (or instrument) error. They are similar in principle to cold kits for ^99m^Tc labelling, are well-accepted by technologists and laboratory staff, do not have high associated consumables costs, and do not require a hot cell environment. As with ^68^Ga-labelled peptides produced by synthesis units, they require quality control by well-established instant thin layer chromatography methods, but are, overall, superior in terms of generator efficiency (for the ^68^Ge/^68^Ga generator with which they are compatible) and simplicity for the user. Several PET/CT facilities in Sydney and Perth attest to the efficiency, high yield and ease of use of kit technology in providing ^68^Ga-PSMA-11 for routine clinical prostate cancer imaging [57]. Other kit formulations of ^68^Ga-PSMA are in preclinical [58] and early clinical development [59]. This evolution of simple kits will be instrumental in the ongoing widespread adoption of ^68^Ga-PSMA PET/CT imaging in staging and restaging of prostate cancer.

## 2. ^68^Ga PSMA PET/CT in Recurrent Disease

**Figure 1:**
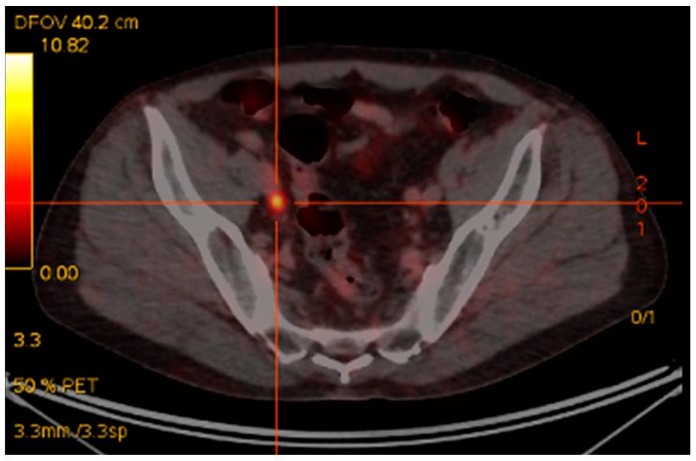
Rising PSA post-prostatectomy. PSA 0.9 ng/mL. CT unremarkable. ^68^Ga-PSMA-avid right pelvic lymph node.

The earliest, and most extensive, experience with ^68^Ga-PSMA PET/CT imaging has been in the most common clinical scenario of biochemical relapse post-definitive primary therapy [60]. Up until the advent of ^68^Ga-PSMA PET/CT the mainstay of imaging included CT +/− bone scintigraphy and in some cases MRI [20]. The largest retrospective study of 1007 patients, reported detection rates for ^68^Ga-PSMA-11 PET/CT of 79.5%, in the setting of biochemical recurrence [61]. A recent meta-analysis revealed detection rates with ^68^Ga-PSMA PET/CT of 58% in patients with PSA between 0.2–1.0 ng/mL, 76% for PSA between 1 and 2 ng/mL and 95% for PSA > 2.0 ng/mL [62]. These findings reflect our own single institutional dataset utilizing ^68^Ga-PSMA I&T in 150 consecutive patients, where we have seen PSMA-avid disease in the setting of biochemical relapse in 25% of patients with PSA < 0.5 ng/mL. 67% of patients with PSA 0.5–1.5 ng/mL and in 92% of patients with PSA > 1.5 ng/mL [63]. Importantly, ^68^Ga-PSMA-I&T PET/CT reveals, in many cases, metastatic disease that is considered occult on CT, as demonstrated in Figure 1. In our dataset, statistically significant differences were seen between recurrent disease demonstrated by ^68^Ga-PSMA I&T and diagnostic contrast CT detection rates [63]. When compared with histological diagnosis, specificities of up to 100% have been detected in pre-surgical nodal assessment prior to nodal salvage surgery using ^68^Ga-PSMA [64,65]. A recent prospective Australian multi-center trial has shown that ^68^Ga-PSMA PET/CT leads to a change in management intent in 62% of patients with biochemical relapse, based upon the PSMA PET/CT scan result [66]. A separate study of 131 patients showed ^68^Ga-PSMA HBED PET/CT had a clinical impact in 76% of patients imaged [67].

^68^Ga-PSMA PET/CT has been shown in multiple studies to be superior to choline-based PET/CT imaging in the setting of biochemical relapse [53,68,69]. Choline PET/CT imaging is now effectively no longer offered clinically in Australia for this indication. Fluorinated PSMA derivatives appear to show equivalence in the setting of mCRPC to that of ^68^Ga-PSMA ligands [50,51,70,71], but are not yet routinely available, and do not have a true theranostic therapeutic pair.

On the strength of the growing body of evidence in this setting of biochemical relapse post definitive primary therapy [60,61,62,63,64,65,66,67,72] the use of ^68^Ga-PSMA PSMA PET/CT been incorporated into commonly accepted imaging algorithms in Australia such as the evidence-based Western Australian Health Diagnostic Imaging Pathways [21]. This clinical acceptance is despite the lack of reimbursement in Australia for ^68^Ga-PSMA PET/CT imaging.

## 3. ^68^Ga-PSMA PET/CT in Primary Disease

**Figure 2:**
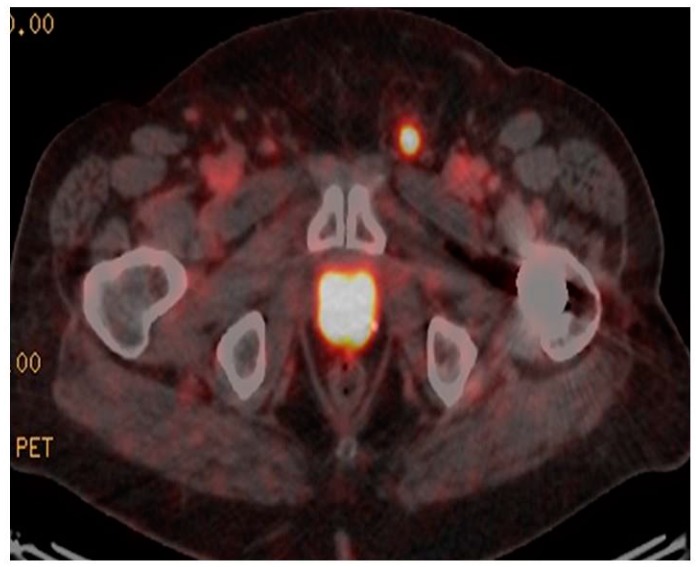
Staging ^68^Ga-PSMA PET/CT. Gleason 4 + 5. PSA 19 ng/mL. Intense ^68^Ga-PSMA avid primary disease in prostate with ^68^Ga-PSMA avid superficial left inguinal node metastasis.

^68^Ga-PSMA PET/CT has recently been investigated as a potential staging modality in primary prostate cancer. Given that cellular PSMA expression has been shown to correlate with PSA and Gleason score [73] and ^68^Ga-PSMA PET/CT has been shown to be superior to standard staging modalities, such as CT, the use of ^68^Ga-PSMA PET/CT would seem to be a logical clinical extension in staging of primary disease. Early data appear to confirm this contention, with several studies [74,75], including our own [76], showing high rates of detection of CT occult early metastatic disease (Figure 2). In our recent study there was very good correlation between PSA and Gleason score and the chance of PSMA-avid metastatic disease. This finding correlates with other studies indicating a role in intermediate-to-high risk primary prostate cancer, with often occult nodal, and occasionally osseous, metastases identified. In assessment of potential bone metastases, ^68^Ga-PSMA PET/CT has been shown to be superior to bone scintigraphy in several studies [77,78].

In assessment of intra-prostatic tumor ^68^Ga-PSMA-11 PET/CT has been compared with multi-parametric MRI, the current standard method for intra-prostatic tumor localization. Interestingly, just as with ^68^Ga-PSMA PET/CT, multiparametric MRI, which is accepted as the most sensitive imaging method for assessing the prostate gland itself [21], is also not reimbursed in Australia. Giesel et al. revealed concordance between positive findings on each modality [79]. Combined ^68^Ga-PSMA-11 PET/CT/MRI was analyzed in 53 patients with intermediate- and high-risk prostate cancer [80], and indicated a superior accuracy of the hybrid approach than with either modality alone. The sensitivities and specificities were 76% and 97%, for hybrid ^68^Ga-PSMA-11 PET/CT/MRI; 58% and 82% for multi-parametric MRI (*p* = 0.003); and 64% and 94%, for ^68^Ga-PSMA-11 PET/CT.

The greatest clinical importance of ^68^Ga-PSMA PET/CT in the staging of primary prostate cancer is the resulting change in management intent in one-fifth of all patients imaged [66]. The prognostic implication of treatment replanning in these patients is substantial. It is hoped that this will be reinforced by the outcome of the ProPSMA study (ACTRN12617000005358), which is currently recruiting in Australia, and a number of studies in the United States (e.g., NCT02611882 and NCT03388346).

## 4. New Clinical Applications

### 4.1. ^68^Ga-PSMA PET/CT and PET/MRI Guided Biopsy

**Figure 3:**
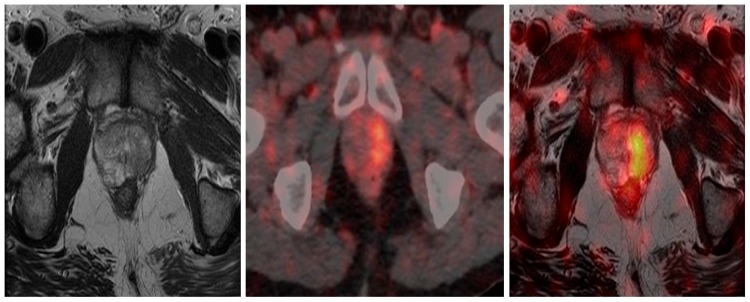
Primary prostate cancer. PIRADS 4/5 left lobe of prostate on multiparametric MRI. Fused to ^68^Ga-PSMA PET/CT images for MRI in-bore guided targeted biopsy.

The high sensitivity of ^68^Ga-PSMA PET/CT, coupled with the increased expression in higher grade tumors, guided intra-prostatic biopsy has shown in several small studies that ^68^Ga-PSMA avidity correlates well with gross tumor volume as detected by multiparametric MRI and voxel based determinants directly matched to histopathological specimens [81,82]. This correlation supports the concept of utilizing these data to potentially reduce sampling error and improve diagnostic accuracy of targeted biopsy. Current prostatic biopsy is often performed blind, although MRI directed biopsy based on Prostate Imaging Reporting and Data System (PI-RADS) is being increasingly utilized [83,84,85,86]. The addition of ^68^Ga-PSMA PET/MRI may further improve the utility and accuracy of this technique and direct biopsy to the most relevant area within the prostate. This is exquisitely demonstrated in Figure 3.

### 4.2. ^68^Ga-PSMA PET/CT-Directed Surgery and Radiotherapy

The visualization of previously unappreciated PSMA-avid small lymph nodes in the pelvis, in both primary staging of prostate cancer and in the setting of biochemical recurrence post-definitive primary therapy, has raised interest in extended or targeted primary lymphadenectomy at time of primary prostatic surgery or as salvage lymphadenectomy. The results, however, of ^68^Ga-PSMA PET/CT targeted nodal surgery, either in the primary or salvage setting, have been relatively poor, with an increase in morbidity and no significant improvement in biochemical remission [65,87,88]. Although the technique shows high specificity (>90%), the sensitivity is only moderate (60–80%), thus the nodes visualized resemble the tip of the iceberg. Micro-metastatic, or very small volume metastatic nodal disease, will be unlikely to be detected even with the resolving capacity of the most modern PET/CT imaging technology. 

Attempts at improving surgical detection by probe techniques, similar to those described with sentinel node biopsy, have been attempted, to improve lymphadenectomy both in the primary and salvage settings. Although data are limited, the use of a gamma probe to detect ^111^In-PSMA injected pre-operatively appears promising in detecting PSMA-avid occult small volume/micro-metastatic nodal disease [89,90,91,92]. This technique also exemplifies the utility of the I&T PSMA compound, which allows successful binding of other radio-isotopes to allow this novel radio-guided surgical approach.

**Figure 4:**
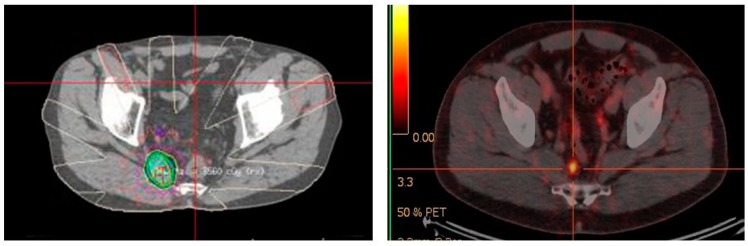
Targeted radiotherapy to recurrent ^68^Ga-PSMA avid right pre-sacral lymph node. June 2015.

The results with ^68^Ga-PSMA PET/CT directed external beam radiotherapy in the primary and salvage setting have been encouraging. Several studies have shown a change in radiotherapy planning from 20–60% of patients imaged with ^68^Ga-PSMA PET/CT prior to external beam radiotherapy [93,94,95]. ^68^Ga-PSMA PET/CT also has a role post-definitive radiotherapy in detecting site of biochemical relapse. In an Australian study of 419 men treated with external beam radiotherapy, ^68^Ga-PSMA PET/CT identified all cases of biochemical relapse [96]. This allows potential salvage therapy if the recurrence is outside the previous radiotherapy field, as was the case for the patient in Figure 4. A negative ^68^Ga-PSMA PET/CT scan in the setting of biochemical recurrence also appears to have prognostic implications for salvage radiotherapy. In a study of 164 Australian men, a negative ^68^Ga-PSMA PET/CT showed treatment response benefit compared with patients with a positive ^68^Ga-PSMA PET/CT scan [97]. Although seemingly counter-intuitive, this again reinforces the likely scenario that very small volume/micro-metastatic nodal recurrence is more likely to be amenable to pelvic radiotherapy than disease that is visible on ^68^Ga-PSMA PET/CT. By this stage the chance of disease having spread outside the confines of the area of the salvage pelvic radiotherapy field, is likely to be high. Whether systemic therapy, combined with either chemotherapy or targeted radiopeptide therapy, together with salvage pelvic radiotherapy can improve response rates in this setting has yet to be elucidated. The long-term outcome of altering treatment planning based on ^68^Ga-PSMA PET/CT has not yet been addressed in any large longitudinal studies.

### 4.3. Monitoring Treatment Response

**Figure 5:**
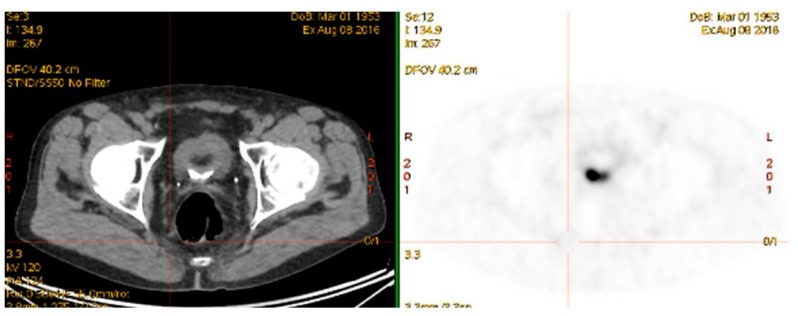
Monitoring response of targeted radiotherapy by ^68^Ga-PSMA PET/CT August 2016. (Previous ^68^Ga-PSMA avid right pre-sacral lymph node treated June 2015—see Figure 4).

Serial ^68^Ga-PSMA PET/CT scans have been used to assess the intermediate term outcomes of radiotherapy in recurrent nodal disease [98,99] (Figure 5). These studies show reductions in SUV in most lesions treated, indicating response, however, the responses based on ^68^Ga-PSMA PET/CT may take several months to be fully realized [99]. Therapeutic response of chemotherapy based on ^68^Ga-PSMA PET/CT imaging has been poorly studied with only a single paper showing a potential role of this imaging modality with monitoring the effectiveness of docetaxel chemotherapy [100]. This is, however, a growing area of interest as ^68^Ga-PSMA PET/CT imaging starts taking over the traditional role that CT and bone scintigraphy has had in monitoring chemotherapeutic response in prostate cancer patients [27]. The poor correlation of RECIST to recognized measures of evaluating molecular responses to cancer treatments, such as the European Organization for Research and Treatment of Cancer (EORTC) criteria has been documented [101]. There is a consensus movement within the nuclear medicine community to harmonize quantitative methods to more accurately compare tumor response to treatment, such as with standard uptake values, and using established criteria such as those found in the EORTC [102], and the PERCIST criteria [103]. More specific definitions for interpreting and evaluating responses ^68^Ga-PSMA PET/CT have also been described [104]. These molecular based evaluation systems will likely replace the antiquated RECIST criteria for assessing therapeutic response in the era of targeted therapies and molecular imaging.

### 4.4. ^68^Ga-PSMA PET/CT Guided Theranostic Pair Therapy with ^177^Lu-PSMA or ^225^Ac-PSMA

Given that cancer is a molecular disease characterized by alterations in molecular epitopes, we can target specific biomarkers, such as PSMA, for imaging. Such imaging agents can be radiolabelled with diagnostic gamma emitters or positron emitters and their theranostic pair with beta and alpha radiometals such as lutetium-177 and actinium-225 for therapeutic purposes. The power of this theranostic paradigm has been well demonstrated in gastroentero-pancreatic neuroendocrine tumors (GEP-NETs) over many years [105,106,107,108,109,110,111] and recently confirmed in the randomized controlled NETTER-1 trial which showed a five-fold improvement in response with ^177^Lu-DOTATATE (Lutathera™) compared with conventional treatment [112]. The theranostic principle allows us “to see what we treat and treat what we see” [113]. The use of molecular imaging agents to target specific therapy is directly analogous to the use of Herceptin in patients with breast cancer where treatment is only given on the prior demonstration of the specific HER-2 receptor on tumor cells [114]. ^68^Ga-PSMA PET/CT imaging performs the same gate keeping function by in vivo demonstration of the upregulation of the PSMA receptor in prostate cancer.

**Figure 6:**
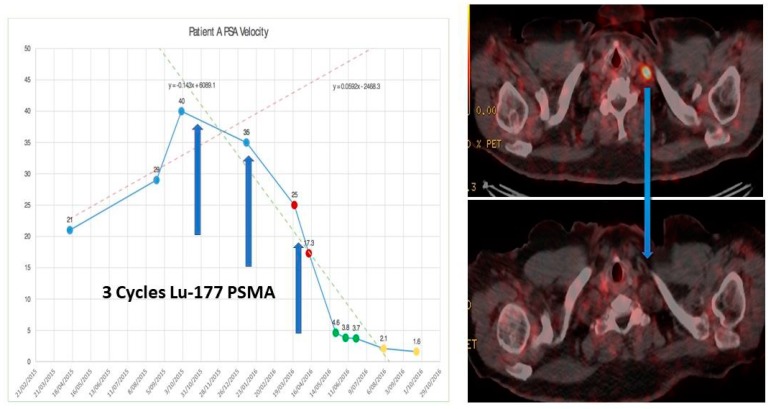
Before (**top**) and after (**bottom**) 3 cycles of ^177^Lu-PSMA therapy for progressive metastatic castrate resistant prostate cancer. [X axis—time in months/; Y axis—PSA (μg/L)].

^68^Ga-PSMA PET/CT has been used over the last 4–5 years in its theranostic capacity to guide therapy using the labelled therapeutic radiopharmaceutical ^177^Lu-PSMA (Figure 6). This targeted radiopeptide therapy has been shown to decrease PSA levels, objectively decrease tumor volumes and tumor activity and improve progression free survival in 40–70% of mCRPC patients who have failed previous treatment modalities including chemotherapy [2,115,116,117,118,119,120]. This has been shown to be a well-tolerated treatment with minimal acute or medium term side-effects, similar to our experience with ^177^Lu-DOTATATE in NET’s. More recently targeted alpha therapy ^225^Ac-PSMA has also been shown, in much smaller studies, to also decrease PSA levels, tumor volumes and tumor activity in patients who have failed ^177^Lu-PSMA [3,121]. A more pronounced effect on salivary gland function has been identified following ^225^Ac-PSMA therapy. Longer term toxicities, if any, are yet to be determined.

## 5. Conclusions

^68^Ga-PSMA PET/CT imaging has become in a relatively short period of time, the gold standard for restaging recurrent prostate cancer in clinical centers in which this imaging modality is available. It is likely to become the standard imaging modality in the staging of intermediate-to-high risk primary prostate cancer. The potential to guide therapy, and to facilitate more accurate prostatic biopsy is being explored. In the theranostic paradigm, ^68^Ga-PSMA PET/CT imaging is critical for detecting PSMA-avid disease which may then respond to targeted ^177^Lu-PSMA or ^225^Ac-PSMA therapies. The ^68^Ga-PSMA PET/CT is being investigated as a method to monitor the therapeutic response to all treatment modalities.
